# Associations between ventricular boundary shift integral and composite cognitive domains across the alzheimer’s disease continuum

**DOI:** 10.1038/s41598-026-39465-9

**Published:** 2026-03-18

**Authors:** Hamide Nasiri, Hannaneh Azimizonuzi, Farbod Khosravi, Seyedeh Fahimeh Hosseini, Alireza Khoshrou, Setareh Rezakhani, Reyhaneh Badri, Danial Kazemi, Negar Toorani, Kobra Soltanikhadiv, Sadra Behrouzieh, Seyed Mohammad Amin Alavi, Shayan Shakeri, Mahsa Mayeli, Farshad Shahkarami

**Affiliations:** 1 Student Research Committee, School of Medicine, Zanjan University of Medical Sciences, Zanjan, Iran; 2 Inventor Member, Iran Representative Office of the International Federation of Inventors’ Associations (IFIA), Tehran, Iran; 3 Shahid Beheshti University of Medical Sciences, Tehran, Iran; 4 Mechanical Engineering Department, Sharif University of Technology, Tehran, Tehran, Iran; 5 Student Research Committee, Mashhad University of Medical Sciences, Mashhad, Razavi Khorasan Iran; 6 Electrical Engineering Department, Islamic Azad University of Najaf Abad, Najaf Abad, Isfahan Iran; 7 Faculty of Sports and Health Sciences, University of Tehran, Tehran, Tehran, Iran; 8 Student Research Committee, Isfahan University of Medical Sciences, Isfahan, Isfahan Iran; 9 School of Modern Science, Islamic Azad University Medical Branch of Tehran, Tehran, Iran; 10 School of Cognitive Neuroscience, Institute for Cognitive Science Studies, Tehran, Tehran, Iran; 11 School of Medicine, Tehran University of Medical Sciences, Tehran, Tehran, Iran; 12 Faculty of Medicine, Ahvaz Jundishapur University of Medical Sciences, Ahvaz, Khuzestan Iran; 13 Department of Medical Genetics, School of Medicine, Shiraz University of Medical Sciences, Shiraz, Iran; 14 School of Medicine, Tehran University of Medical Sciences, Tehran, Iran; 15 Present Address: Department of Internal Medicine, School of Medicine, Tehran University of Medical Sciences, Tehran, Iran

**Keywords:** Alzheimer’s disease, Mild cognitive impairment, Brain atrophy, Magnetic resonance imaging, Boundary shift integral, Biomarkers, Neurology, Neuroscience

## Abstract

Cognitive impairment is hallmark of Alzheimer’s disease (AD). Although structural MRI has consistently demonstrated widespread brain atrophy in AD, the comparative utility of different imaging sequences for predicting cognitive decline remains uncertain. The Boundary Shift Integral (BSI), a longitudinal measure quantifying brain volume change over time, provides a dynamic alternative to conventional cross-sectional volumetric measures and could more sensitively reflect cognition-related neurodegeneration. This study investigated the associations between BSI-derived atrophy and domain-specific cognitive performance across the AD continuum. Participants (Cognitively Normal [CN] = 155, Mild Cognitive Impairment [MCI] = 283, AD = 100) underwent comprehensive cognitive testing, and composite scores were computed for memory, executive function, and language domains. BSI metrics for the whole brain, ventricles, and bilateral hippocampi were derived from serial MRI scans acquired at baseline and 12-month follow-up and were directly compared with conventional T1-weighted volumetric measures. Linear regression models were used to examine associations between imaging markers and domain-specific cognitive scores, adjusting for relevant covariates. In the MCI group, BSI measures demonstrated significant associations with all cognitive domains, consistently outperforming traditional volumetric measures. Whole-brain and ventricular BSI were significantly associated with memory, executive function, and language, while hippocampal BSI showed particularly robust associations with memory. In the AD group, BSI again exhibited more consistent associations with cognitive performance than volumetrics, especially for whole-brain and right hippocampal measures. No significant associations were observed in the CN group. Direct comparisons confirmed that BSI provides enhanced sensitivity to cognition-relevant structural changes, particularly in early and prodromal stages of AD. BSI measures offer superior sensitivity in detecting early structural brain changes associated with cognitive decline. These findings support the utility of BSI as a dynamic and clinically relevant tool for early detection and longitudinal monitoring of the disease.

## Introduction

Alzheimer’s disease (AD) is the most prevalent form of dementia, accounting for approximately 60% to 80% of cases worldwide^1,2^. By 2050, its global prevalence is expected to more than double, posing a substantial public health burden^3^. The pathophysiology of AD is strongly associated with the accumulation of amyloid-beta (Aβ) plaques and neurofibrillary tangles^4^. These neurodegenerative changes predominantly affect brain regions responsible for memory processing, particularly the hippocampus^5–8^. Consequently, memory impairment is one of the earliest and most defining symptoms of AD, with deficits in short-term memory and executive function becoming increasingly pronounced as the disease progresses^9,10^.

Executive functions encompass a broad spectrum of cognitive processes, including planning, working memory, attention control, and cognitive flexibility, which are essential for the performance of daily activities. These functions are often disrupted in patients with mild cognitive impairment (MCI) and AD^11–13^. Structural and functional alterations in the inferior frontal junction, a region integral to cognitive control processes such as task switching and working memory, have been strongly associated with executive dysfunction in AD^14^. Beyond memory and executive function deficits, language impairments may also emerge early in AD, sometimes preceding significant memory decline^15^. Research indicates that difficulties in lexical retrieval and semantic processing often occur during the prodromal stages of the disease^16–18^. These early deficits underscore the potential of language function as a diagnostic marker, offering valuable insights into disease progression and severity^18,19^.

The boundary shift integral (BSI) has emerged as a measure of quantifying whole-brain atrophy rates in AD^20^. This semi-automated method leverages serial three-dimensional (3D) MRI scans to measure volumetric changes by assessing boundary displacements in specific brain structures over time^21,22^. BSI has been widely used to quantify neurodegeneration and atrophy across various neurological disorders, including multiple sclerosis and Huntington’s disease^23–30^, and dementia^31^. Generalized BSI (gBSI) is a recently developed measure incorporating probabilistic segmentation, providing enhanced sensitivity to atrophy progression in AD while reducing sample size requirements in clinical trials^21^. Building on these advancements, a multi-time-point BSI method was subsequently developed to improve longitudinal atrophy estimation^32^. Another study found associations between white matter hyperintensities and BSI in cognitively normal (CN) individuals^33^. These findings underscore the utility of BSI in assessing neurodegeneration in ADand support its potential for early detection and disease monitoring, including tracking hippocampal atrophy in AD^21,33,34^. Despite the extensive body of literature employing BSI and related volumetric techniques, most prior studies^19,35^ have focused on global or regional atrophy rates without systematically examining their domain-specific cognitive correlates across diagnostic stages.

To address these gaps, we investigated the associations between whole-brain, ventricular, and hippocampal BSI measures and composite cognitive scores in domains of executive function, memory, and language in individuals across AD continuum. Using these composite cognitive scores allows our results to be directly comparable across major AD cohorts. We hypothesized that greater structural changes based on BSI measures will be significantly associated with poorer cognitive outcomes across these domains. 

## Methods and materials

### Participants and study design

The data for this study were obtained from the ADNI database (http://adni.loni.usc.edu/), a public-private partnership established in 2003 under the leadership of Principal Investigator Dr. Michael W. Weiner. The primary objective of ADNI is to evaluate whether a combination of serial magnetic resonance imaging (MRI), positron emission tomography (PET), biological markers, and clinical and neuropsychological assessments can reliably track the progression of MCI and early AD. Participants, aged 55 to 90, were required to undergo neuroimaging, lumbar punctures, and longitudinal follow-up assessments, with detailed inclusion and exclusion criteria outlined in previous publications. Key exclusion criteria included a Hachinski Ischemic Score greater than 4, use of unapproved medications, recent changes in permitted medications, a Geriatric Depression Scale score of 6 or higher, and fewer than six years of education or equivalent work experience.

Participants were classified into three groups: CN, individuals with MCI, and those with AD. Classification criteria were as follows. Normal controls reported no memory complaints, whereas participants with MCI or AD were required to have subjective memory concerns. Mini-Mental State Examination (MMSE) scores ranged from 24 to 30 for cognitively normal and MCI participants, and from 20 to 26 for those with AD (inclusive ranges). The Clinical Dementia Rating (CDR) was 0 for normal controls, 0.5 for MCI (with a mandatory memory box score ≥ 0.5), and 0.5–1.0 for AD.

Memory performance was assessed using the delayed recall of one story from the Logical Memory II subscale of the Wechsler Memory Scale–Revised (maximum score: 25). Education-adjusted cutoffs were applied: for cognitively normal participants, scores were ≥ 9 for ≥ 16 years of education, ≥ 5 for 8–15 years, and ≥ 3 for ≤ 7 years. Corresponding cutoffs for MCI and AD were ≤ 8, ≤4, and ≤ 2, respectively^36^. A detailed description of subject selection and cohort demographics is available elsewhere^37^. For this analysis, we included participants with available datasets of BSI measures, composite cognitive scores, and clinical measures.

### Composite scores assessment

The study employed the ADNI-MEM and ADNI-EF composite scores, which are psychometrically robust measures of memory and executive function, respectively, to identify individuals exhibiting significant cognitive alterations. ADNI-MEM and ADNI-EF were previously described by Crane et al. (2012) and Gibbons et al. (2012), respectively^38,39^. The ADNI-MEM score is derived from participants’ performance across multiple cognitive assessments, including the MMSE, Alzheimer’s Disease Assessment Scale–Cognitive Subscale (ADAS-Cog), Rey Auditory Verbal Learning Test (RAVLT), and Logical Memory tests^40–43^. Similarly, ADNI-EF factor scores are calculated using observed scores from a range of executive function tests, including Digit Symbol Coding and Digit Span, Trail Making Tests A and B, category fluency tasks (animals and vegetables), and the Clock Drawing Test^44–48^. Language function was evaluated using the ADNI-LAN composite score^49^, which integrates performance on the Boston Naming Test, MMSE, ADAS-Cog, and the Montreal Cognitive Assessment (MoCA)^50,51^.

### Neuroimaging

Preprocessed imaging data were downloaded from ADNI. Based on ADNI methodology, brain regions were automatically delineated using multiatlas propagation and segmentation (MAPS)^52,53^, with manual correction performed when required^22^. Following registration to the MNI atlas, the ventricular regions were delineated using semi-automated intensity-based segmentation techniques^54^. Longitudinal scan alignment was performed using a nine-degree-of-freedom registration algorithm^55^. 

Differential bias correction (DBC) was applied to correct intensity inhomogeneity between baseline and follow-up scans^56.^.

BSI values were calculated using an established preprocessing pipelines, including both classic-BSI and k-means normalized BSI (KN-BSI) methods^54^.These approaches incorporate tissue-specific intensity normalization and automated parameter selection to enhance sensitivity to longitudinal atrophy. Because ADNI is a multicenter study, all T1-weighted MRI scans were acquired using ADNI’s standardized 3D MPRAGE protocol across scanner vendors (GE, Siemens, Philips). The ADNI MRI core implemented calibration procedures using phantom imaging and site-specific corrections to ensure geometric and intensity consistency across imaging sites. 

All MRI scans underwent rigorous quality control using the Design Rule Check (DRC) procedure. Images were evaluated for anatomical coverage, motion artifacts, and longitudinal consistency. Only scans with quality ratings ≤ 3 (Good to Borderline-Acceptable) were included. Only accelerated metrics, scans acquired using ADNI’s standardized accelerated T1-weighted MRI protocol designed to reduce motion artifacts and optimize signal-to-noise ratio, were included in the current study^57^. The final BSI values used in this analysis were obtained directly from the ADNI database, where image preprocessing and BSI computation were performed by the ADNI imaging core.

### Statistical analysis

Data analysis was performed using Python, incorporating the following packages: pandas (v2.1.4), statsmodels (v0.14.0), pingouin (v0.5.4), SciPy (v1.12.0), and numpy (v1.26.4). The Shapiro-Wilk test was used to assess the normality of variable distributions. Outlier values, defined as those exceeding ± 2 standard deviations from the mean, were removed and replaced with the corresponding group mean. Continuous variables are reported as mean (SD), while categorical variables are presented as frequencies. Group comparisons for normally distributed continuous variables were conducted using one-way ANOVA, whereas non-normally distributed variables were analyzed using the Kruskal-Wallis test. The chi-square test was applied to evaluate the distribution of categorical variables across groups.

Post hoc analyses were conducted using Tukey’s HSD test for significant ANOVA results (*p* < 0.05) and Dunn’s test with Bonferroni correction for significant Kruskal-Wallis test results.

To assess longitudinal changes in cognitive performance, we compared paired baseline and 12th month follow-up scores for memory, executive function, and language domains within each diagnostic group. Normality of the difference distributions was evaluated using the Shapiro-Wilk test. Depending on the distribution, either a paired t-test (for normally distributed differences) or a Wilcoxon signed-rank test (for non-normally distributed differences) was applied.

Linear regression models were employed within each diagnostic group to investigate the predictive value of BSI-derived metrics and volumetric changes on composite cognitive scores, controlling for age, gender, and years of education. Statistical significance was set at *p* < 0.05. All p-values reported for regression analyses were corrected for False Discovery Rate (FDR) using the Benjamini-Hochberg procedure (FDR-adjusted p) to account for multiple comparisons.

## Results

A total of 586 participants met the eligibility criteria: 176 CN, 305 MCI, and 105 AD. Demographic characteristics of participants are summarized in Table 1. The three diagnostic groups did not differ significantly in age (overall *p* = 0.154, Kruskal–Wallis test) or years of education (overall *p* = 0.227, Kruskal–Wallis test). However, there was a significant difference in sex distribution across groups (*p* = 0.039, χ^2^ test). Both CDR-SB and MMSE scores showed significant group differences (both *p* < 0.001, Kruskal–Wallis test).

**Table 1:** Demographic characteristics of participants.

	CN (*N* = 176)	MCI (*N* = 305)	AD (*N* = 105)	Comparison	P value	Overall P value
Age, year	72.76 ± 5.99	71.92 ± 6.83	73.2 ± 6.42	CN vs. MCI	0.516	0.154^a^
				CN vs. AD	0.516	
				MCI vs. AD	0.182	
Gender						
Female	96 (54.5)	138 (45.25)	42 (40)			0.039^b^
Male	80 (45.5)	167 (54.75)	63 (60)			
Education, year	16.59 ± 2.52	16.27 ± 2.62	16.08 ± 2.51	CN vs. MCI	0.407	0.227^a^
				CN vs. AD	0.309	
				MCI vs. AD	0.477	
CDR-SB	0.13 ± 0.37	1.38 ± 1.07	3.95 ± 1.23	CN vs. MCI	**< 0.001**	**< 0.001** ^**a**^
				CN vs. AD	**< 0.001**	
				MCI vs. AD	**< 0.001**	
MMSE	28.982 ± 1.23	27.83 ± 1.97	24.53 ± 2.31	CN vs. MCI	**< 0.001**	**< 0.001** ^**a**^
				CN vs. AD	**< 0.001**	
				MCI vs. AD	**< 0.001**	

Significant values are in [bold].

CN, cognitively normal; MCI, mild cognitive impairment; AD, Alzheimer’s disease; MMSE, Mini-Mental State Examination; CDR-SB, Clinical Dementia Rating Sum of Boxes; ^a^ Kruskal-Wallis test; ^b^ Chi square test.

Whole-brain normalized BSI values increased stepwise from CN to MCI to AD participants (*p* < 0.001, ANOVA). Similarly, ventricular BSI values were significantly elevated in MCI and AD compared with CN (*p* < 0.001, Kruskal–Wallis test). At the regional level, both right and left hippocampal BSI values were significantly higher in MCI and AD (overall *p* < 0.001 for both, ANOVA/Kruskal–Wallis) (Table 2).

**Table 2:** Comparison of boundary shift integral metrics among groups.

	CN (*N* = 176)	MCI (*N* = 305)	AD (*N* = 105)	Comparison	P value	Overall P value
Whole brain K mean Normalized-BSI	6.73 ± 6.35	9.07 ± 7.28	15.71 ± 7.33	CN vs. MCI	0.003	< 0.001^a^
				CN vs. AD	< 0.001	
				MCI vs. AD	< 0.001	
Ventricular BSI	1.38 ± 1.51	1.97 ± 1.8	3.57 ± 1.75	CN vs. MCI	0.001	< 0.001^b^
				CN vs. AD	< 0.001	
				MCI vs. AD	< 0.001	
Right hippocampal BSI	0.03 ± 0.05	0.04 ± 0.06	0.09 ± 0.05	CN vs. MCI	0.032	< 0.001^b^
				CN vs. AD	< 0.001	
				MCI vs. AD	< 0.001	
Left hippocampal BSI	0.03 ± 0.05	0.05 ± 0.06	0.09 ± 0.05	CN vs. MCI	0.009	< 0.001^a^
				CN vs. AD	< 0.001	
				MCI vs. AD	< 0.001	

CN, cognitively normal; MCI, mild cognitive impairment; AD, Alzheimer’s disease; BSI, Boundary Shift Integral; ^a^ ANOVA test; ^b^ Kruskal-Wallis test.

Whole-brain volume showed significant decline across diagnostic groups (overall *p* = 0.01, ANOVA). Ventricular volume progressively increased from CN to AD (overall *p* < 0.001, Kruskal–Wallis test). Pronounced hippocampal atrophy was evident bilaterally. Right hippocampal volume decreased from CN to AD (overall *p* < 0.001, Kruskal–Wallis), and left hippocampal volume followed a similar pattern (overall *p* < 0.001, ANOVA) (Table 3).

**Table 3:** Comparison of T1-MRI volumetrics among groups.

	CN (*N* = 176)	MCI (*N* = 305)	AD (*N* = 105)	Comparison	P value	Overall P value
Whole brain volume	1060.48 ± 87.94	1061.81 ± 93.97	1029.9 ± 99.22	CN vs. MCI	0.813	0.01^a^
				CN vs. AD	**0.002**	
				MCI vs. AD	**0.008**	
Ventricular volume	34.25 ± 14.43	36.29 ± 17.9	45.4 ± 16.01	CN vs. MCI	0.369	< 0.001^b^
				CN vs. AD	**< 0.001**	
				MCI vs. AD	**< 0.001**	
Right hippocampal volume	2.77 ± 0.32	2.66 ± 0.37	2.38 ± 0.33	CN vs. MCI	**0.003**	< 0.001^b^
				CN vs. AD	**< 0.001**	
				MCI vs. AD	**< 0.001**	
Left hippocampal volume	2.68 ± 0.31	2.56 ± 0.36	2.25 ± 0.32	CN vs. MCI	**< 0.001**	< 0.001^a^
				CN vs. AD	**< 0.001**	
				MCI vs. AD	**< 0.001**	

Significant values are in [bold].

CN, cognitively normal; MCI, mild cognitive impairment; AD, Alzheimer’s disease; ^a^ ANOVA test; ^b^ Kruskal-Wallis test.

Composite scores differed significantly among the three groups at both baseline and 12-month follow-up (Table 4). At baseline, CN participants exhibited higher memory, executive function, and language composite scores compared with MCI and AD groups (all overall *p* < 0.001, Kruskal–Wallis/ANOVA). Memory performance showed a stepwise decline across groups (*p* < 0.001 for all pairwise contrasts). Similar gradients were observed for executive function (*p* < 0.001) and language performance (*p* < 0.001). At the 12-month follow-up, memory, executive function, and language scores decreased in MCI and AD.

**Table 4:** Comparison of composite cognitive scores among groups.

	CN (*N* = 176)	MCI (*N* = 305)	AD (*N* = 105)	Comparison	*P* value	Overall *P* value
Baseline						
Memory	0.82 ± 0.43	0.33 ± 0.49	-0.53 ± 0.36	CN vs. MCI	< 0.001	< 0.001^a^
				CN vs. AD	< 0.001	
				MCI vs. AD	< 0.001	
Executive function	0.75 ± 0.49	0.5 ± 0.52	0.04 ± 0.47	CN vs. MCI	< 0.001	< 0.001^b^
				CN vs. AD	< 0.001	
				MCI vs. AD	< 0.001	
Language	0.78 ± 0.41	0.51 ± 0.44	0.11 ± 0.45	CN vs. MCI	< 0.001	< 0.001^a^
				CN vs. AD	< 0.001	
				MCI vs. AD	< 0.001	
12-month follow-up						
Memory	0.91 ± 0.45	0.34 ± 0.65	-0.73 ± 0.39	CN vs. MCI	< 0.001	< 0.001^a^
				CN vs. AD	< 0.001	
				MCI vs. AD	< 0.001	
Executive function	0.78 ± 0.45	0.51 ± 0.54	-0.07 ± 0.56	CN vs. MCI	< 0.001	< 0.001^a^
				CN vs. AD	< 0.001	
				MCI vs. AD	< 0.001	
Language	0.88 ± 0.43	0.56 ± 0.51	-0.07 ± 0.43	CN vs. MCI	< 0.001	< 0.001^a^
				CN vs. AD	< 0.001	
				MCI vs. AD	< 0.001	

CN, cognitively normal; MCI, mild cognitive impairment; AD, Alzheimer’s disease; ^a^ Kruskal-Walli’s test; ^b^ ANOVA test.

Longitudinal analyses of composite scores revealed distinct trajectories across diagnostic groups over the follow-up period (Table 5). Among CN participants, significant improvements were observed in memory (*p* < 0.001, paired t-test) and language performance (*p* = 0.001). No significant change was detected in executive function (*p* = 0.142). In contrast, the MCI group demonstrated significant increase in language scores (*p* = 0.017). The AD group exhibited statistically significant decline in memory performance and language abilities.

**Table 5:** Longitudinal changes in cognitive domains across diagnostic groups.

	CN (*N* = 176)			MCI (*N* = 305)			AD (*N* = 105)		
	Baseline	Follow-up	*P* value	Baseline	Follow-up	*P* value	Baseline	Follow-up	*P* value
Memory	0.80 ± 0.42	0.88 ± 0.43	**< 0.001** ^**a**^	0.33 ± 0.48	0.34 ± 0.64	0.614 ^a^	-0.46 ± 0.38	-0.67 ± 0.37	**< 0.001** ^**a**^
Executive function	0.71 ± 0.48	0.75 ± 0.45	0.142 ^a^	0.50 ± 0.51	0.53 ± 0.51	0.187 ^a^	0.12 ± 0.46	0.02 ± 0.56	0.052 ^a^
Language	0.76 ± 0.40	0.85 ± 0.43	**0.001** ^**a**^	0.52 ± 0.43	0.57 ± 0.49	**0.017** ^**a**^	0.25 ± 0.44	0.05 ± 0.41	**< 0.001** ^**a**^

Significant values are in [bold].

CN, cognitively normal; MCI, mild cognitive impairment; AD, Alzheimer’s disease; ^a^ Paired t test.

**Fig. 1:**
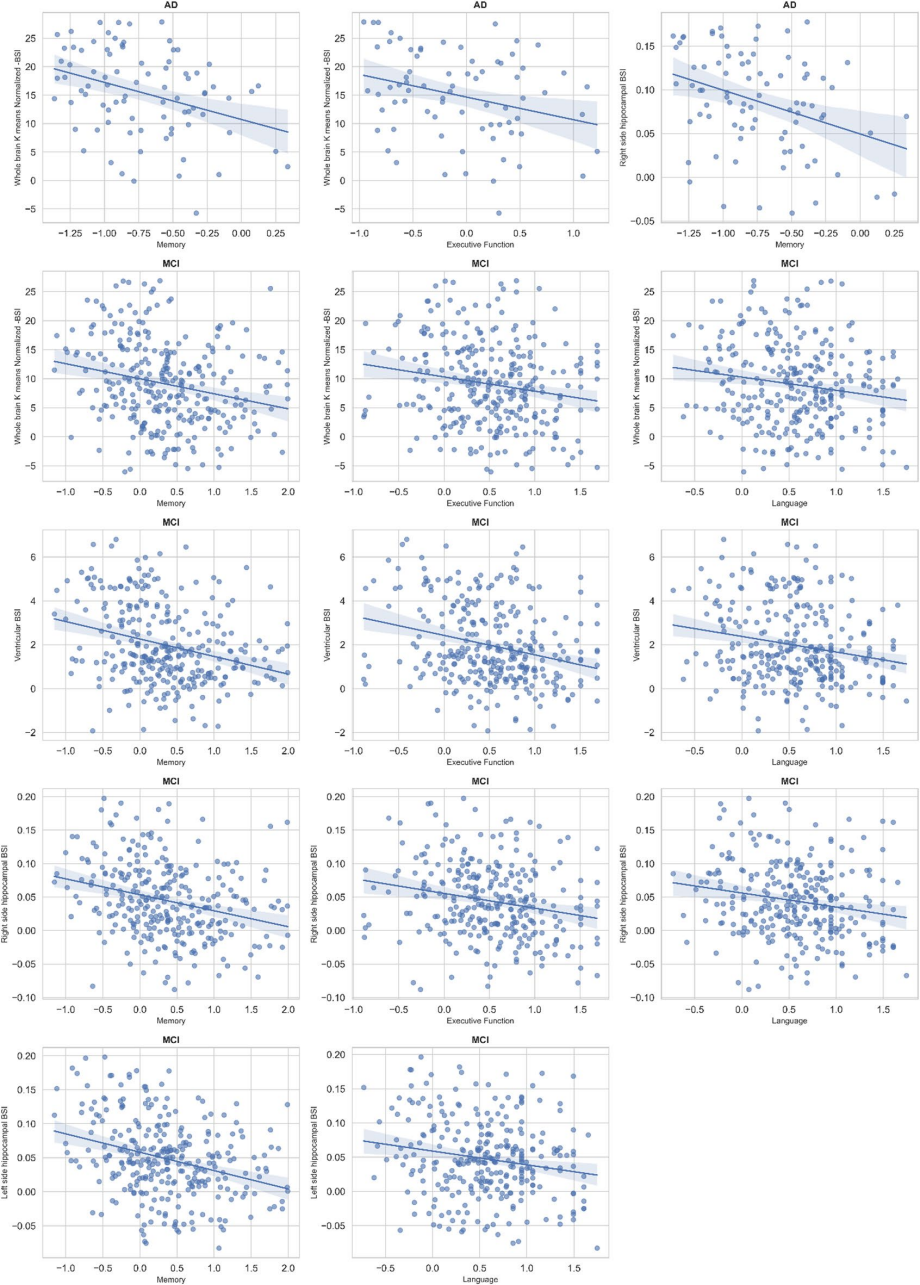
Associations between boundary shift integral (BSI) indices and cognitive performance across diagnostic groups. Scatter plots illustrate significant associations between normalized whole-brain, ventricular, and hippocampal BSI measures and cognitive composite scores, including memory, executive function, and language, across cognitively normal (CN), mild cognitive impairment (MCI), and Alzheimer’s disease (AD) groups. Regression models were adjusted for age, gender, and education. Each data point represents an individual participant, and regression lines denote the direction of association. All p-values were corrected for multiple comparisons using the false discovery rate method (FDR-adjusted p). Significant negative correlations were observed primarily in the MCI and AD groups, indicating that higher atrophy rates were associated with poorer cognitive performance.

Associations between baseline BSI measures and cognitive composite scores are presented in Table 6; Fig. 1. All regression models accounted for age, gender, and education. In CN participants, none of the BSI indices showed significant associations with memory, executive function, or language performance (FDR-adjusted *p* > 0.05 for all). Among individuals with MCI, higher whole-brain normalized BSI was significantly associated with poorer memory (β = − 0.20, FDR-adjusted *p* < 0.001) and executive function (β = − 0.16, FDR-adjusted *p* = 0.005). Similar negative associations were observed for ventricular (β = − 0.21, FDR-adjusted *p* < 0.001) and hippocampal BSI metrics. Specifically, right and left hippocampal BSI values were negatively associated with memory (β = − 0.21 and − 0.26, respectively; both FDR-adjusted *p* < 0.001) and language performance (β = − 0.13, FDR-adjusted *p* = 0.022 for both). Whole-brain BSI (β = − 0.38, FDR-adjusted *p* = 0.003) and right hippocampal BSI (β = − 0.32, FDR-adjusted *p* = 0.009) associated with memory decline, respectively, whereas executive and language functions showed nonsignificant associations (FDR-adjusted *p* > 0.05) in AD participants.

**Table 6:** Linear regression results between boundary shift integral and harmonized composite cognitive scores.

	CN (*N* = 176)			MCI (*N* = 305)			AD (*N* = 105)		
	Adjusted R^2^	Standardized β	FDR adjusted p value	Adjusted R^2^	Standardized β	FDR adjusted p value	Adjusted R^2^	Standardized β	FDR adjusted p value
Dependent variable: whole brain K means normalized -BSI									
Memory	0.18	-0.1	0.156	0.22	-0.2	**< 0.001**	0.14	-0.38	**0.003**
Executive function	0.14	0	0.951	0.21	-0.16	**0.005**	0.09	-0.29	**0.039**
Language	0.14	-0.05	0.488	0.2	-0.14	**0.022**	0.01	-0.2	0.274
Dependent variable: ventricular BSI									
Memory	0.19	-0.12	0.118	0.19	-0.21	**< 0.001**	0.08	-0.26	0.058
Executive function	0.15	-0.02	0.951	0.21	-0.23	**< 0.001**	0.05	-0.15	0.364
Language	0.15	-0.12	0.219	0.21	-0.15	**0.022**	-0.01	-0.14	0.464
Dependent variable: right hippocampal BSI									
Memory	0.22	-0.17	0.073	0.21	-0.21	**< 0.001**	0.1	-0.32	**0.009**
Executive function	0.15	-0.05	0.951	0.2	-0.16	**0.006**	0.03	0.07	0.536
Language	0.13	-0.1	0.257	0.2	-0.13	**0.022**	-0.04	-0.08	0.631
Dependent variable: left hippocampal BSI									
Memory	0.19	-0.15	0.073	0.23	-0.26	**< 0.001**	0	-0.06	0.577
Executive function	0.14	-0.01	0.951	0.18	-0.11	0.051	0	0.09	0.536
Language	0.14	-0.15	0.168	0.2	-0.13	**0.022**	-0.05	0.04	0.734

Significant values are in [bold].

CN, cognitively normal; MCI, mild cognitive impairment; AD, Alzheimer’s disease; BSI, Boundary Shift Integral.; FDR: False Discovery Rate.

**Fig. 2:**
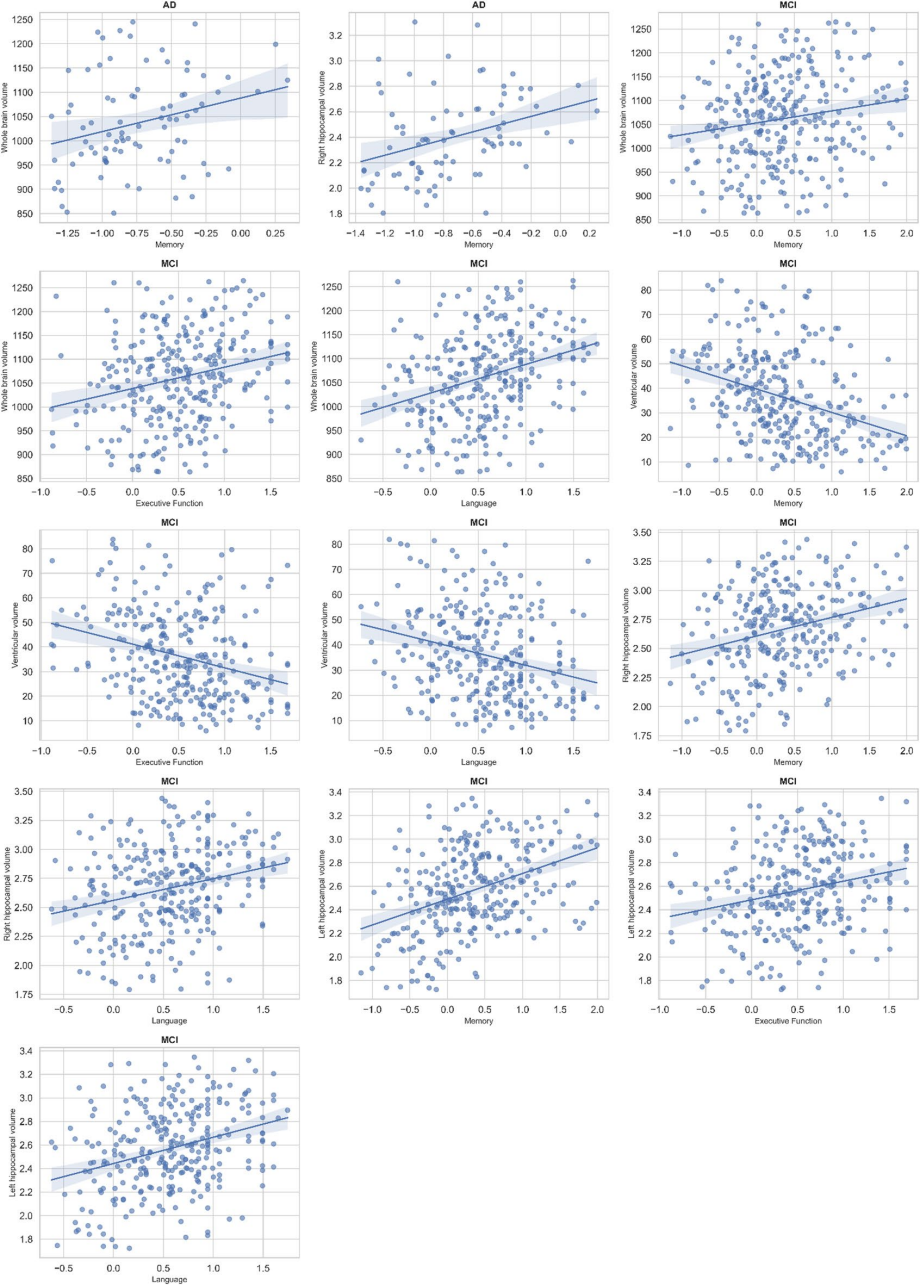
Associations between baseline volumetric MRI measures and cognitive performance across diagnostic groups. Scatter plots show significant associations between whole-brain, ventricular, and hippocampal volumes and cognitive composite scores (memory, executive function, and language) in CN, MCI, and AD participants. Linear regression models were adjusted for age, gender, and education, and all p-values were corrected using the false discovery rate method (FDR-adjusted p). Larger brain and hippocampal volumes were associated with better memory and language performance, whereas increased ventricular volume correlated with poorer cognitive outcomes. The strength of these associations was most pronounced in the MCI group.

Moreover, associations between baseline volumetric MRI measures and cognitive composites are summarized in Table 7; Fig. 2. In CN participants, none of the volumetric indices were significantly associated with memory, executive, or language function (FDR-adjusted *p* > 0.05 for all). In contrast, significant associations emerged in MCI group. Larger whole-brain (β = 0.17, FDR-adjusted *p* = 0.012), right hippocampal (β = 0.26, FDR-adjusted *p* < 0.001), and left hippocampal volumes (β = 0.38, FDR-adjusted *p* < 0.001) were associated with better memory performance. Similarly, greater left hippocampal (β = 0.26, FDR-adjusted *p* < 0.001) and whole-brain volumes (β = 0.31, FDR-adjusted *p* < 0.001) were linked to superior language abilities, while reduced ventricular volume predicted higher executive and memory scores (β = − 0.23 and − 0.21; FDR-adjusted *p* ≤ 0.005). Whole-brain volume correlated with memory (β = 0.30, FDR-adjusted *p* = 0.028) in AD group. No significant associations were observed for executive or language composites in the AD group (FDR-adjusted *p* > 0.05).

**Table 7:** Linear regression results with T1 MRI volumetric measures as a function of composite cognitive scores.

	CN (*N* = 176)			MCI (*N* = 305)			AD (*N* = 105)		
	Adjusted R^2^	Standardized β	FDR adjusted p value	Adjusted R^2^	Standardized β	FDR adjusted p value	Adjusted R^2^	Standardized β	FDR adjusted p value
Dependent variable: whole brain volume									
Memory	0.18	-0.06	0.955	0.17	0.17	**0.012**	0.05	0.3	**0.028**
Executive function	0.15	0.04	0.86	0.18	0.14	**0.036**	0.01	0.09	0.483
Language	0.14	0.15	0.339	0.25	0.31	**< 0.001**	-0.04	0.06	0.622
Dependent variable: ventricular volume									
Memory	0.17	-0.07	0.955	0.2	-0.23	**< 0.001**	0.02	-0.14	0.254
Executive function	0.14	0.06	0.86	0.2	-0.21	**0.005**	0	-0.1	0.483
Language	0.12	-0.02	0.827	0.21	-0.17	**0.009**	-0.01	-0.12	0.447
Dependent variable: right hippocampal volume									
Memory	0.18	0.02	0.955	0.19	0.26	**< 0.001**	0.09	0.3	**0.028**
Executive function	0.15	-0.01	0.94	0.16	0.11	0.07	0.03	-0.11	0.483
Language	0.14	0.07	0.485	0.19	0.2	**0.001**	-0.01	0.21	0.326
Dependent variable: left hippocampal volume									
Memory	0.18	0	0.955	0.27	0.38	**< 0.001**	0.05	0.26	0.050
Executive function	0.15	-0.07	0.86	0.17	0.14	**0.036**	0.02	-0.13	0.483
Language	0.14	0.08	0.485	0.24	0.26	**< 0.001**	-0.02	0.17	0.326

Significant values are in [bold].

CN, cognitively normal; MCI, mild cognitive impairment; AD, Alzheimer’s disease; FDR: False Discovery.

## Discussion

This study examined the associations between whole brain, hippocampal, and ventricular atrophy and composite cognitive scores in patients across the AD continuum. Our findings demonstrate a progressive increase in whole brain atrophy, ventricular enlargement, and hippocampal atrophy from CN to MCI to AD, with the most pronounced structural changes in the AD group. Notably, we found strong associations between BSI metrics and cognitive decline in MCI and AD, including language, memory, and executive function.

Among individuals with MCI, BSI metrics showed the strongest and most consistent associations with memory, executive function, and language scores. In particular, hippocampal BSI exhibited robust associations with memory performance, consistent with the central role of hippocampal atrophy in AD pathology, indicating that structural hippocampal changes are among the earliest features of MCI. In the AD group, BSI also captured significant cognitive associations, most notably with memory and executive function, although effect sizes were somewhat lower than in MCI, possibly reflecting ceiling effects in late-stage neurodegeneration or reduced variability in performance due to more severe global impairment. BSI’s predictive value persisted in this more advanced cohort, underscoring its potential for disease staging and monitoring beyond the MCI stage. These results highlight the differential impact of global and regional atrophy on cognitive function, emphasizing the value of BSI as a neuroimaging marker for disease progression in AD. They are consistent with previous studies regarding BSI’s association with cognitive decline and hippocampal atrophy in AD^58^.

Our findings suggest that neurodegenerative changes are most pronounced in the transitional stage of AD, impacting multiple cognitive domains. However, after FDR correction, hippocampal BSI no longer showed significant associations with cognitive function, suggesting that in later disease stages, whole-brain and ventricular atrophy may be stronger indicators of cognitive impairment. The observed shift from hippocampal atrophy to more global atrophy as a predictor of cognitive decline aligns with the established model of MCI progression into AD. In early stages, particularly MCI, the hippocampal degeneration is a key pathological feature, explaining its strong correlation with memory function. However, as the disease progresses, widespread cortical atrophy and ventricular expansion become more pronounced, potentially making whole-brain and ventricular BSI more reliable markers of cognitive decline. This aligns with previous studies reporting that ventricular enlargement and whole-brain atrophy accelerate with increasing AD severity, eventually overshadowing the impact of hippocampal atrophy in later stages^59–61^.

Ventricular enlargement is a well-established marker of brain atrophy and has been associated with white matter changes, impaired interstitial fluid (ISF) and CSF dynamics, and dysfunction of the glymphatic system, all processes implicated in AD progression^62–66^.In our study, BSI-derived measures of ventricular expansion provide a quantitative assessment of these structural changes over time. Increased ventricular volume often reflects combined gray and white matter loss, which may compromise the efficiency of waste clearance mechanisms, including glymphatic transport. Impaired clearance can lead to the accumulation of neurotoxic proteins such as β-amyloid, thereby linking morphometric changes to underlying pathophysiological processes. By integrating longitudinal ventricular BSI with cognitive outcomes, our findings suggest that ventricular enlargement may not only serve as a sensitive imaging biomarker of structural degeneration but also indirectly reflect disruptions in brain clearance mechanisms that contribute to cognitive decline. These results reinforce the utility of combining morphometric and functional insights to understand disease progression and identify early indicators of AD.

While previous studies have primarily focused on regional BSI metrics, our findings suggest the utility of whole-brain BSI as a valuable tool for monitoring AD progression. For example, a study examining global and regional atrophy rates over 36 months in CN, MCI, and AD patients, found that while whole-brain atrophy rates remained stable, hippocampal atrophy and ventricular enlargement rates accelerated in MCI and AD, particularly in MCI patients who later progressed to AD^31^. This suggests that hippocampal and ventricular BSI may serve as a more sensitive marker of early-stage neurodegenerative changes, whereas whole-brain BSI remains an important metric for tracking overall disease progression. We also performed a direct comparison between conventional T1-weighted MRI measures and BSI, showing that BSI metrics consistently exhibit stronger associations with cognitive scores in individuals with AD or MCI, highlighting its potential as a robust imaging biomarker. This finding contributes to the expanding body of evidence supporting BSI as a sensitive marker of neurodegeneration, with demonstrated utility across a range of neurological conditions, including AD, multiple sclerosis, and frontotemporal dementia^22,26,58,67–71^.

Our findings align with previous research highlighting the critical role of neurodegeneration and brain atrophy, particularly in the entorhinal cortex (ERC) and hippocampus in cognitive decline and functional impairment in AD. One study similarly reported that BSI-derived hippocampal and temporal horn volume changes can predict conversion from MCI to AD^70^. Interestingly, in that study, the temporal horn expansion rate was a stronger predictor than hippocampal atrophy, suggesting that BSI may serve as a sensitive tool for detecting early AD-related neurodegeneration. Another study comparing whole-brain BSI with ERC and hippocampal atrophy rates in AD and normal aging found approximately a 2.5-fold greater whole-brain BSI and more than a fivefold greater ERC/hippocampal atrophy in AD^70^. While ERC and hippocampal measures were more sensitive for tracking AD progression, a combination of ERC and ventricular BSI most effectively distinguished AD from controls^70^. These findings support previous reports indicating that ERC atrophy rates exceed those of the hippocampus in AD, reinforcing the notion that AD pathology originates in the ERC^72^.

The lateralized associations observed in this study highlight the anatomical and functional asymmetry of the hippocampal subfields. Previous research has demonstrated the particular vulnerability of the left hippocampus to atrophy associated with episodic verbal memory decline in prodromal AD, reflecting the left hemisphere’s dominance in language-related processing^73–76^. Conversely, the right hippocampus has been implicated in visuospatial and nonverbal memory functions^75–77^, such as Memory free recall, face-name association, and short-term maintenance of object-location relationships^78^. Our results are consistent with these observations. For instance, one small-scale 2014 study found that left hippocampal volume loss correlated with lower MMSE and AVLT scores, whereas right hippocampal atrophy was associated with reduced AVLT performance exclusively in AD group. Moreover, reduced activities of daily living and naming ability were linked to left hippocampal volume reduction only in the AD patients^79^. We similarly found strong correlations between left hippocampal BSI and cognitive scores, particularly within the memory and language domains, whereas right hippocampal BSI showed a stronger association with executive function. Interestingly, we also observed a weak but significant association between right hippocampal BSI and memory performance in CN individuals. To the best of our knowledge, this has not been previously reported and may suggest that subtle hippocampal atrophy could serve as an early biomarker of cognitive vulnerability, even before the onset of clinical symptoms. However, given the modest effect size and the absence of other significant associations in this group, further validation in larger, independent cohorts is warranted. These lateralized findings support accumulating evidence that the left and right hippocampus have distinct functional specializations, and that neurodegenerative changes in AD may manifest asymmetrically, with corresponding cognitive consequences that reflect the unique roles of each hippocampal hemisphere.

## Limitations

First, our study relies on ADNI cohort, which may not fully represent the general population^80^. For instance, the gender imbalance across groups could introduce potential biases in the findings. Second, although our analyses were longitudinal and included both baseline and follow-up assessments, the observational nature of the study limits our ability to draw definitive causal inferences regarding the relationship between BSI measures and cognitive function. While our findings suggest that BSI-derived atrophy indices may reflect ongoing neurodegenerative changes associated with cognitive decline, future longitudinal studies with larger and more diverse cohorts, extended follow-up durations, and multimodal imaging approaches are warranted to confirm the temporal sequence of these associations and to evaluate the predictive value of BSI measures for cognitive deterioration over time. Further research should also explore whether combining BSI metrics with other imaging techniques (such as PET or fMRI) enhances predictive accuracy for AD progression. Additionally, investigating other brain regions, such as the entorhinal cortex, may provide a more comprehensive understanding of structural changes in AD. Moreover, recent studies have suggested that individuals with positive amyloid and tau in their CSF show accelerated brain atrophy and cognitive decline, with significant whole-brain, hippocampal, and cortical thinning, and have a higher risk of progressing to MCI or dementia^81,82^. Therefore, incorporating genetic, vascular, and lifestyle factors into future analyses could offer deeper insights into individual variability in atrophy progression and cognitive decline.

## Conclusion

This study underscores the utility of BSI metrics as sensitive and reliable indicators of cognitive decline and neurodegeneration across the AD continuum, with particular application in the MCI stage. By comparing BSI to conventional T1-derived volumetric measures, we found that BSI demonstrated stronger associations with domain-specific cognitive scores, especially in individuals with MCI. In the MCI group, whole-brain and ventricular BSI metrics were significantly associated with memory, executive function, and language performance, often surpassing the strength and extent of associations seen with corresponding volumetric indices. Notably, hippocampal BSI, particularly in the left hemisphere, exhibited robust associations with memory and executive function, reinforcing its potential as an early marker of cognitive vulnerability. These findings suggest that BSI captures dynamic, cognition-relevant changes with greater sensitivity than static volumetric measures, offering a more nuanced reflection of early neurodegenerative processes.

Taken together, these results support the integration of BSI-derived regional and global atrophy metrics into multimodal assessment frameworks. By enhancing sensitivity to domain-specific cognitive decline, particularly in the prodromal phase, BSI holds promise as a powerful neuroimaging biomarker for improving early diagnosis, disease staging, and stratification in clinical trials. Future longitudinal and multimodal studies are warranted to validate its prognostic value and explore its integration with molecular and fluid biomarkers for comprehensive disease characterization across the AD continuum.

## Data Availability

The datasets analyzed in this study are available from the ADNI repository upon submission of a request and after approval. Custom code used for data analysis can be obtained from the corresponding author upon reasonable request.
